# Biochanin A induces anticancer effects in SK-Mel-28 human malignant melanoma cells via induction of apoptosis, inhibition of cell invasion and modulation of NF-κB and MAPK signaling pathways

**DOI:** 10.3892/ol.2021.12724

**Published:** 2021-04-12

**Authors:** Peng Xiao, Bowen Zheng, Jiaming Sun, Jia Yang

Oncol Lett 14: 5989-5993, 2017; DOI: 10.3892/ol.2017.6945

Following the publication of the above paper, a concerned reader drew to the Editor's attention that several figures (specifically, Figs. 2, 3 and 4) contained apparent anomalies, including repeated patternings of data within the same figure panels.

After having conducted an independent investigation in the Editorial Office, the Editor of *Oncology Letters* has determined that this paper should be retracted from the Journal on account of a lack of confidence concerning the originality and the authenticity of the data. The authors were asked for an explanation to account for these concerns, but the Editorial Office never received any reply. The Editor regrets any inconvenience that has been caused to the readership of the Journal.

